# Physical activity and disability in patients with noncardiac chest pain: a longitudinal cohort study

**DOI:** 10.1186/s13030-020-00185-9

**Published:** 2020-06-30

**Authors:** Joanne Castonguay, Stéphane Turcotte, Richard P Fleet, Patrick M Archambault, Clermont E Dionne, Isabelle Denis, Guillaume Foldes-Busque

**Affiliations:** 1 School of Psychology, Université Laval, Pavillon Félix-Antoine-Savard, 2325 Allée des Bibliothèques, Québec, QC G1V 0A6 Canada; 2 Centre intégré de santé et de services sociaux de Chaudière-Appalaches, 143 Rue Wolfe,, Lévis, QC G6V 3Z1 Canada; 3 Département de médecine familiale et de médecine d’urgence, Université Laval, Pavillon Ferdinand-Vandry, 1050 Avenue de la Médecine, Québec, QC G1V 0A6 Canada

**Keywords:** Noncardiac chest pain, Physical activity, Disability, Protective effect

## Abstract

**Background:**

Noncardiac chest pain (NCCP) is one of the leading reasons for emergency department visits and significantly limits patients’ daily functioning. The protective effect of physical activity has been established in a number of pain problems, but its role in the course of NCCP is unknown. This study aimed to document the level of physical activity in patients with NCCP and its association with NCCP-related disability in the 6 months following an emergency department visit.

**Methods:**

In this prospective, longitudinal, cohort study, participants with NCCP were recruited in two emergency departments. They were contacted by telephone for the purpose of conducting a medical and sociodemographic interview, after which a set of questionnaires was sent to them. Participants were contacted again 6 months later for an interview aimed to assess their NCCP-related disability.

**Results:**

The final sample consisted of 279 participants (57.0% females), whose mean age was 54.6 (standard deviation = 15.3) years. Overall, the proportion of participants who were physically active in their leisure time, based on the Actimètre questionnaire criteria, was 22.0%. Being physically active at the first measurement time point was associated with a 38% reduction in the risk of reporting NCCP-related disability in the following 6 months (*ρ* = .047). This association remained significant after controlling for confounding variables.

**Conclusions:**

Being physically active seems to have a protective effect on the occurrence of NCCP-related disability in the 6 months following an emergency department visit with NCCP. These results point to the importance of further exploring the benefits of physical activity in this population.

## Background

Noncardiac chest pain (NCCP) is one of the leading reasons for emergency department visits, accounting for 2 to 5% of all visits [1]. Furthermore, a large proportion of patients with NCCP repeatedly consult various physicians and medical specialists [2, 3]. In the United States, the annual costs associated with these visits are estimated between $8 and $13 billion [3–6].

NCCP poses a heavy burden on patients, which is compounded by the fact that the symptoms become recurrent in the long term in up to 90% of cases [7–10]. Although the cardiovascular prognosis of NCCP following an emergency department visit is generally favourable, this symptom significantly and persistently limits the daily functioning, quality of life and ability to engage in physical activity for 20 to 60% of patients [3, 8, 10, 11].

Patients with NCCP are more than twice as likely to be physically inactive during their leisure time than the general population (23% vs. 10%; 13). However, the picture of these patients’ level of activity is incomplete as the rate of transportation- and work-related physical activity has yet to be documented in this population. The observed high level of physical inactivity in adult with NCCP is potentially fraught with biopsychosocial consequences, since, in addition to being a risk factor for the development of a number of serious health problems, it may contribute to the maintenance of NCCP and of the resulting disability [12, 13]. Indeed, a lower pain tolerance threshold has been reported in physically inactive individuals [14], which could contribute to the development and maintenance of NCCP. Furthermore, inactivity may result in physical deconditioning that increases the likelihood of experiencing physical symptoms, such as NCCP [15, 16]. Lastly, for many patients with NCCP, physical inactivity results from a fear of triggering a heart problem, despite the absence of a diagnosis to justify such a fear [17, 18]. Being inactive, they do not have the opportunity to confront these fears, which could contribute to their maintenance [19]. Yet, these fears are associated with the perpetuation and exacerbation of NCCP and of the resulting disability [20, 21].

On the other hand, physical activity appears to have a protective and potentially therapeutic effect in a number of pain problems [22–28]. Indeed, it can raise the pain tolerance threshold through gradual habituation to pain symptoms [12, 29]. It also reduces anxiety sensitivity [30, 31], the fear of anxiety-related physical sensations and their consequences [32], a factor associated with NCCP-related disability [33]. Physical activity is also associated with a 35% reduction in heart-focused anxiety, defined as the fear of cardiovascular sensations and their anticipated negative consequences [34, 35]. This is highly relevant to NCCP as heart-focused anxiety explains much of the efficacy of interventions for NCCP [36, 37]. By acting on several biopsychosocial mechanisms associated with the exacerbation and maintenance of NCCP, physical activity might lower the risk of experiencing NCCP-related disability.

A better understanding of this link would make it possible to determine if increased physical activity can limit the development of NCCP-related disability. In order to fully understand the role of physical activity, it is essential to consider the influence of biopsychosocial factors that have been associated with the development and maintenance of NCCP and the related disability. The main biological factors associated with NCCP are sex, age and medical conditions [10, 20, 38]. Studies and biopsychosocial models of NCCP or unexplained medical symptoms link social support and heart-focused anxiety with the development and maintenance of NCCP and its consequences [20, 34, 36, 39]. While psychological distress and anxiety sensitivity are also relevant constructs, it appears to be through their association with heart-focused anxiety [33, 36, 40, 41]. This goal of this study was to deepen our knowledge of the link between physical activity and NCCP-related disability.

## Objectives and hypotheses

The first objective of this study was to document the level of physical activity in patients with NCCP. The second objective consisted in exploring the association between patients with NCCP level of physical activity at the time of an emergency department visit with NCCP and NCCP-related disability in the following 6 months.

As previously reported in the literature, it was expected that the level of physical activity in patients with NCCP would be lower than in the general population. It was also expected that a higher level of physical activity would be associated with a lower likelihood of NCCP-related disability at six-month follow-up.

## Methods

### Design and setting

This prospective, longitudinal cohort study was carried out between April 2015 to March 2016 in two emergency departments of the Centre intégré de santé et de services sociaux de Chaudière-Appalaches (CISSS-CA; the University affiliated hospital Hôtel-Dieu de Lévis and the Centre Paul-Gilbert). The CISSS-CA ethics board authorized this project (CER-1314-022). This article was written following the STROBE guidelines [42].

### Participants

To be included in the study, adult patients (18 years of age or older) had to present with NCCP at low risk for mortality or for cardiovascular involvement, which was defined as nontraumatic chest pain without radiologically apparent cause that could explain the chest pain, without new malignant arrhythmia and a *Modified Thrombolysis in Myocardial Infarction score* of two or less [43, 44]. They also had to be able to read and understand French and have completed the study’s measures of NCCP and physical activity. The exclusion criteria were as follows: a terminal illness; a severe communication problem that would have interfered with the administration of the interview and questionnaires; and a condition that could have invalidated the interview, such as being in a psychotic state or having a major cognitive impairment.

### Procedure

This study is part of a larger longitudinal research project. All participants corresponding to the eligibility criteria were selected to constitute the sub-sample of this study. Recruitment was carried out prospectively by a team of research nurses, who assessed the eligibility of all the patients who came to the two emergency departments with a complaint of chest pain. Eligible patients were approached on site or by phone. A member of the research team contacted the consenting, eligible participants within 4 weeks of their consultation to conduct a telephone interview for the purpose of assessing NCCP-related disability and gathering sociodemographic and medical history data. A set of questionnaires was then sent to the participants by mail or via the PIANO secured Web portal [45]. The participants were contacted again 6 months later for an interview aimed at assessing NCCP-related disability. A reminder procedure and prize draws were put in place to maximize the questionnaire return rate.

### Measures

#### Sociodemographic and medical interview

This interview was used to record the participants’ age and biological sex and gather other informations, such as their level of formal education, employment status, civil status, family income, weight and height to calculate body mass index as well as their medical history (diagnoses and interventions).

***Chest Pain Interview*** (adapted from Eslick and Talley [7] and from Jonsbu, Dammen [46]). This interview was used to assess average NCCP intensity and the number of chest pain episodes in the last 6 months. It was also used to assess NCCP-related disability, the main dependent variable in this study, in the previous 6 months with four items that respectively assess social, family, work and physical activity sphere of functioning using four points likert scales (i.e., 1. no impact; 2. mild impact; 3. moderate impact; or 4. severe impact [47];). As proposed by Jonsbu, Dammen [46] patients reporting moderate to severe impact on one of the items were considered to present significant NCCP-related disability.

#### The Actimètre questionnaire [48]

This questionnaire was used to determine an individual’s level of physical activity on an annual basis. It was developed from international criteria from Kesaniemi, Danforth [49] and public health recommendations regarding the minimum level of physical activity required to achieve health benefits [50, 51]. The score obtained places the individuals at one of the following four levels of physical activity, defined on the basis of the energy expenditure index (EEI), which is expressed in kcal/kg/week: active (≥ 14 EEI), moderately active (≥ 7 to < 14 EEIs), somewhat active (> 0 < 7 EEIs) or inactive (0 EEIs). The level of physical activity of an individual considered ‘active’ corresponds to 1000 kcal/week for a 70-kg reference standard individual [48] which is the minimal amount recommended by public health agencies for achieving substantial health benefits [49–51]. The level of physical activity is reported globally and for each constituent subscale: leisure-time, transportation-related and work-related physical activity. The global score is calculated according to an algorithm [48] and reflects the highest activity level reached in a given subscale.

#### MOS social support survey (MOS-SSV [52, 53];)

This instrument was designed to evaluate the perceived social support of patients with chronic conditions. It contains 7 items rated on a 5-point Likert-type scale ranging from 0 (never) to 4 (always). The higher the total score (range: 0 to 28, converted in 0 to 100), the better the perceived support. Like the original version, this French-Canadian adaptation has good internal consistency and temporal stability [52, 53].

#### ***Cardiac Anxiety Questionnaire****(CAQ* [54, 55];

This 4-subscale questionnaire was used to assess heart-focused anxiety (anxiety associated with cardiovascular sensations). The French-Canadian adaptation of the CAQ contains 15 items rated on a Likert-type scale ranging from 0 (never) to 4 (always), for a total score varying between 0 and 60 [55]. Its subscales are fear, avoidance, attention to cardiovascular sensations, and reassurance seeking. The higher the score, the higher the heart-focused anxiety or its components are considered to be. The CAQ has excellent internal consistency (α = 0.88) and excellent divergent and convergent validity [55].

### Analyses

Descriptive statistics were used to describe the final sample. The sociodemographic characteristics of the participants in the final sample and of those who did not complete the study’s main measures were compared using Student’s *t*-tests for continuous variables and chi-square tests for categorical variables. This comparison was done in order to assess the sample’s representativeness. Missing continuous values were substituted with the corresponding item average for the sample.​ Missing categorical values were not imputed.

For analysis, patients were categorized into two groups according to their level of physical activity on the Actimètre questionnaire: the physically active group and the below recommended threshold group which combine the moderately active, somewhat active and inactive categories. The later group was used as the reference category. A log-binomial regression model was used to evaluate the bivariate association between the level of physical activity and the presence of NCCP-related disability in the following 6 months of the emergency department visit with NCCP (dependent variable). This type of analysis is preferable to logistic regression here because it directly yields prevalence ratios rather than an estimate of the risk ratios based on the odds ratio. Indeed, when the prevalence of the dependent variable is greater than 10%, the estimate of the relative risk based on the odds ratio can be inaccurate [56]. The potential confounding variables (age, sex, a history of gastrointestinal or cardiovascular disease, social support education level, smoking status, number of NCCP episodes in previous 6 months, average NCCP intensity and heart-focused anxiety) were added to the log-binomial regression model using the backward selection method. The model’s goodness of fit was verified using the Hosmer-Lemeshow test [57, 58]. All the analyses were performed using IBM SPSS Statistics software, version 23 (IBM Corp., New York), and an alpha level of 0.05.

## Results

### Sample characteristics

**Fig. 1:**
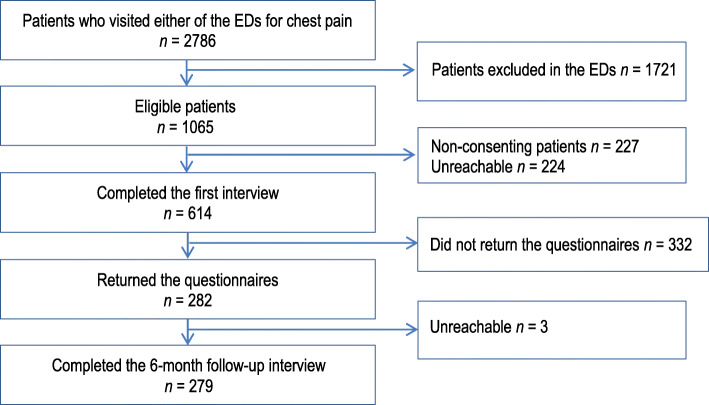
Participant flowchart

Fig. 1 shows the patients flowchart. The final sample consisted of 279 participants (159 females and 120 males) who visited one of the two emergency departments with NCCP. Their mean age was 54.6 (*SD* = 15.3) years. The participants had a significantly higher education level than the patients who were excluded from this study because they did not complete the study’s main measures (physical activity and NCCP-related disability; see Table 1).

**Table 1:** Sociodemographic characteristics

Characteristics	Patients who did not complete the main measures (*n* = 335)	Participants (*n* = 279)	*t* or chi-square	*ρ*
Age, mean (*SD*)	52.2 (16.3)	54.6 (15.3)	1.8	.066
Female, % (*n*)	50.1 (168/335)	57.0 (159/279)	2.9	.091
Civil status: married or common law, % (*n*)	67.1 (222/331)	73.7 (205/278)	3.2	.073
Education > 12 years, % (*n*)	46.4 (153/330)	56.1 (156/278)	5.7	.017
Currently working, % (*n*)	58.9 (195/331)	53.0 (148/279)	2.0	.160
Family income < $60 K, % (*n*)	57.19 (180/315)	56.5 (148/262)	0.0	.874
Body mass index, mean (*SD*)	27.1 (11.1)	27.4 (9.3)	0.3	.774
Current smokers, % (*n*)	21.1 (70/332)	12.9 (36/279)	7.1	.008
NCCP-related disability, % (*n*)	45.4 (149/328)	40.6 (112/276)	1.4	.231
Number of NCCP episodes in the last 6 months, mean (*SD*)	16.3 (41.7)	17.5 (48.0)	−0.3	.751
Average NCCP intensity, mean (*SD*)	5.4 (2.4)	5.2 (2.4)	0.8	.443

*SD* Standard deviation.

### Physical activity and NCCP-related disability

Participants were classified as being physically active in 52.0% (*n* = 145/279) of cases. The breakdown of the participants’ level of physical activity in each category (leisure time, transportation and work) is detailed in Table 2.

**Table 2:** Level of physical activity at the time of the initial visit

Level of physical activity		Initial visit			
		Global	Leisure time	Transportation	Work
Below recommended threshold	Inactive, % (n)	21.9 (61/279)	45.5 (126/277)	78.9 (217/275)	49.3 (135/274)
	Mildly active, % (n)	10.4 (29/279)	17.3 (48/277)	10.9 (30/275)	2.9 (8/274)
	Moderately active, % (n)	15.8 (44/279)	15.2 (42/277)	5.1 (14/275)	11.7 (32/274)
Active, % (n)		52.0 (145/279)	22.0 (61/277)	5.1 (14/275)	36.1 (99/274)

At the time of the initial emergency department visit, 40.6% (*n* = 112/276) of the sample reported NCCP-related disability. This prevalence was 21.5% (*n* = 60/279) in the following 6 months.

### Association between the level of physical activity and NCCP-related disability in the following 6 months

Log-binomial regression using the descending selection method generated a model that included the global level of physical activity and the presence of a history of cardiovascular or gastrointestinal disease. The contribution of these three variables to the risk of reporting NCCP-related disability was significant (see Table 3). Thus, age, sex, social support (MOS-SSV), education level, smoking status, baseline reported NCCP frequency, mean NCCP intensity and heart-focused anxiety (CAQ) were excluded from the final model. The Hosmer-Lemeshow test showed that the final model fit the data well, *ρ* = .843 (> .05).

**Table 3:** Factors associated with NCCP-related disability in the following 6 month

Variables	Bivariate (*n* = 279)			Multivariate (*n* = 279)			
	Prevalence Ratio	95% *CI*	*ρ*	Prevalence Ratio	95% *CI*	Wald Chi-square (*df*)	*ρ*
Age	1.01	1.00–1.03	.075	–	–	–	–
Sex	0.71	0.44–1.15	.163	–	–	–	–
Social support (MOS-SSV)	1.00	0.97–1.03	.944	–	–	–	–
Education level	0.89	0.57–1.40	.624	–	–	–	–
Current smoker	1.03	0.54–2.00	.920	–	–	–	–
Number of NCCP episodes, last 6 months	1.00	1.00–1.00	.736	–	–	–	–
Average NCCP intensity	1.08	0.98–1.19	.121	–	–	–	–
Heart-focused anxiety (CAQ)	1.03	1.01–1.05	<.001	1.02	1.00–1.04	3.04 (1)	.081
Global physical activity: active (*Actimètre questionnaire*)	0.54	0.34–0.86	.009	0.62	0.39–1.00	3.93 (1)	.047
History of gastrointestinal disease	2.16	1.37–3.41	<.001	1.93	1.22–3.05	7.99 (1)	.005
History of cardiovascular disease	1.93	1.24–3.00	.004	1.60	1.03–2.49	4.38 (1)	.036

*CI* Confidence interval, *df* Degrees of freedom, *MOS-SSV* MOS Social Support Survey, *CAQ* Cardiaque anxiety questionnaire

## Discussion

This study assessed the global physical activity level in patients with NCCP and prospectively assessed its potential protective effect on NCCP related disability. Overall, during the year preceding their emergency department visit with NCCP, half (48%) of the sample had not reached the minimum level of physical activity required to achieve health benefits, based on the Actimètre questionnaire criteria (i.e. active, moderately active, somewhat active or inactive; 51). Over one fifth of the sample (21.9%) was considered inactive, which is consistent with results from a previous study [59]. The proportion of participants who were physically active in their leisure time according to the Actimètre questionnaire was 34% lower than in the general population of the same age and region (22.0% vs. 33.3% [60];). Substantial differences were also observed in transportation-related physical activity, as the percentage of inactive study participants for this category was 26% higher than in the general population (78.9% vs. 62.8% [60];). Thus, patients with NCCP are less active than the general population, which confirms the first hypothesis.

As for the second objective, being physically active at the first time point was associated with a 46% reduction in the risk of reporting NCCP-related disability in the following 6 months. This association remained significant, with a 38% reduction of this risk, when potential biopsychosocial confounding variables (age, sex, a history of gastrointestinal or cardiovascular disease, social support education level, smoking status, number of NCCP episodes in previous 6 months, average NCCP intensity and heart-focused anxiety) were introduced into the model, which confirms our hypothesis. Of note, in the final model, a history of cardiovascular or gastrointestinal disease appears to increase the risk of reporting NCCP-related disability in the following 6 months by 60 and 93%, respectively. The observed role of gastrointestinal disease in the increased risk of NCCP-related disability might be explained by its association with general sensitization, a mechanism common to both conditions [38, 61]. Furthermore, several gastrointestinal conditions can cause NCCP, among which gastrointestinal reflux disease is the most frequent [62]. Accordingly, this condition may contribute to the maintenance and increased intensity of NCCP over time which, in turn, could result in NCCP-related disability.

Coherent with a biopsychosocial conceptualization of the effect of physical activity, the absence of heart-focused anxiety in the final model, although surprising, can possibly be explained by its inverse correlation with the level of physical activity [35]. Along the same line, the presence of a history of cardiovascular disease in the final model may play a role in increasing the risk of presenting NCCP-related disability by its association with heart-focused anxiety, a factor known to be central in this condition [63, 64]. In addition, in some cases, pathological mechanisms such as microvascular angina could contribute to the maintenance of NCCP [65] and explain the associations between cardiovascular disease and NCCP-related disability.

Physical activity appears to play a protective role in reducing the risk to suffer from NCCP-related disability. This finding highlights the importance of examining the biopsychosocial factors that can influence participation in physical activity in this population, which is already less active than the general population, and looking into ways to promote physical activity for this population. Promoting physical activity appears a promising biopsychosocial intervention because of its effect on key psychological (e.g. heart-focused anxiety) [32, 33], and biological factors (e.g. cardiovascular disease) [35, 66] associated with the development of NCCP-related disability. In order to globally improve the level of physical activity, the literature on the promotion of physical activity stresses the need to intervene at both the population and individual level through several strategies, based mainly on the cognitive-behavioral approach [67, 68]. These strategies include providing information about health linked with physical activity, goal setting, reinforcement of efforts, self-monitoring of physical activity, etc. [68].

### Limitations

Certain limitations inherent in this study should be borne in mind when interpreting its results. Most of the data were collected by means of self-report questionnaires, which leaves open the possibility of recall and social desirability bias [69]. Ascertaining the level of physical activity retrospectively over the previous year leaves open the possibility of potential fluctuation in the level engaged in at the time of the emergency department visit. However, having data that was gathered for a one-year period enabled us to better account for the seasonal variations and provided an estimate of the general physical activity level. In addition, the possibility of an inverse relationship between the variables of interest, that is the impact of NCCP-related disability on physical activity practice in the following 6 months, cannot be eliminated. Moreover, participants in the final sample differed from those who did not complete the main measures in terms of education level and smoking status. These differences may limit the generalizability of the results and influence the level of physical activity reported. A higher education level is associated with increased rates of moderate-to-vigorous physical activity practice compared to lower education level [70]. The lower proportion of smokers in the final sample indicate that the number of patients considered active may have been overestimated in our sample [71]. However, these variables did not contribute to the explanation of NCCP-related disability in the final model.

## Conclusions

In conclusion, being physically active is associated with a lower probability to suffer from NCCP-related disability in the 6 months following an emergency department visit. These findings point to the importance of further investigating the protective role of physical activity in the course of NCCP. Meanwhile, health care providers involved in the care of NCCP could enhance the benefits of exercise for these patients who otherwise tend to suffer persistently.

## Data Availability

The datasets used and analyzed during the current study are available from the corresponding author on reasonable request.

## Abbreviations

*CI*: Confidence interval
CISSS-CA: Centre intégré de santé et de services sociaux de Chaudière-Appalaches
*df*: Degrees of freedom
EEI: Energy expenditure index
NCCP: Noncardiac chest pain
*SD*: Standard deviation
